# Supplementary material for: Arid5a uses disordered extensions of its core ARID domain for distinct DNA- and RNA-recognition and gene regulation
Source: J Biol Chem. 2024 Jun 10;300(7):107457. doi: 10.1016/j.jbc.2024.107457 (PMC11262183; doi:10.1016/j.jbc.2024.107457)
Supplement: Supplemental Material [file mmc1.docx]

# Supplemental material

# Arid5a uses disordered extensions of its core ARID domain for distinct DNA- and RNA-recognition and gene regulation

Julian von Ehr^1,2^, Lasse Oberstrass^3^, Ege Yazgan^4,5^, Lara Ina Schnaubelt^1^, Nicole Blümel^4^, Francois McNicoll^4^, Julia E. Weigand^3^, Kathi Zarnack^4,5^, Michaela Müller-McNicoll^4,6^, Sophie Marianne Korn^1,7*^ and Andreas Schlundt^1,8*^

^1^Institute for Molecular Biosciences and Biomolecular Resonance Center (BMRZ), Goethe University Frankfurt, Max-von-Laue-Str. 7-9, 60438 Frankfurt, Germany.

^2^IMPRS on Cellular Biophysics, Max-von-Laue-Str. 7-9, 60438 Frankfurt, Germany.

^3^University of Marburg, Department of Pharmacy, Institute of Pharmaceutical Chemistry, Marbacher Weg 6, 35037 Marburg, Germany.

^4^Institute for Molecular Biosciences, Goethe University Frankfurt, Max-von-Laue-Str. 13, 60438 Frankfurt, Germany.

^5^Buchmann Institute for Molecular Life Sciences, Goethe University Frankfurt, Max-von-Laue-Str. 15, 60438 Frankfurt, Germany.

^6^Max-Planck Institute for Biophysics, Max-von-Laue-Str. 9, 60438 Frankfurt, Germany

^7^Department of Biochemistry and Molecular Biophysics, Columbia University, New York, NY 10032, USA.

^8^University of Greifswald, Institute of Biochemistry, Felix-Hausdorff-Str. 4, 17489 Greifswald, Germany.

*To whom correspondence should be addressed: smk2305@cumc.columbia.edu; schlundt@bio.uni-frankfurt.de

## Supplementary Table 1. Overview of DNA oligonucleotides used to generate mutants in the ARID domain. Non-capital letters indicate site of mutation.

| Name | Sequence 5’ → 3’ | Usage |
| --- | --- | --- |
| Gibson_Arid5a_49_fw | CGAGAATCTTTATTTTCAGGGCCGCGAGGAAGAGCAGGAACGGG | Gibson assembly Arid5a ARID in pET-Trx1a backbone |
| Gibson_Arid5a_152rev | GGCTTTGTTAGCAGCCGGATCCCGACCCTTACTATTTGTCATCTTCGCCTTTCAGATGGCG |  |
| Gibson_Arid5a_37_fw | CTTTATTTTCAGGGCATTAGCTTGGAAGATTCGCCTGAAGCTGG | Gibson assembly Arid5a ARID in pET-Trx1a backbone |
| Gibson_Arid5a_183rev | GCAGCCGGATCCCGACCCTTACTATTTCGCTTTCTTCGGACGTTCGGTTGCCCC |  |
| Gibson_pETTrx_TEV_rev | GCCCTGAAAATAAAGATTCTCGAGACCACTGCCAGATCCGGCC | Amplification of pET-Trx for Gibson assembly |
| Gibson_pETTrx_Stop_fw | TAAGGGTCGGGATCCGGCTGCTAACAAAGCCCG |  |
| SDM_E57A_fw | GGAACGGGAAGcgGAGCAAGCGTTTC | Mutation of E57 to alanine |
| SDM_E57A_rev | TGCTCTTCCTCGCGTTCA | Mutation of E57 to alanine |
| SDM_R78A_fw | CCCGATTGAAgcgGTTCCACACCTC | Mutation of R78 to alanine |
| SDM_R78A_rev | GTATGGCGTTCCTTCATG | Mutation of R78 to alanine |
| SDM_R109A_fw | AACTGGTCGTgcgTTGTGGAAGAACGTCTATGAC | Mutation of R109 to alanine |
| SDM_R109A_rev | ACCAGCTCATAGGCACCC | Mutation of R109 to alanine |
| SDM_T125S126A_fw | TCCAGGCTCTgCggCgGCAGCTACGT | Mutation of T125 and S126 to alanine |
| SDM_T125S126A_rev | CTTCCGCCTAATTCGTCATAG | Mutation of T125 and S126 to alanine |
| SDM_K85A_Q86A_fw | CCTCGGCTTTgcggcgATCAACCTGTGGAAAATC | Mutation of K85 and Q86 to alanine |
| SDM_K85A_Q86A_rev | TGTGGAACGCGTTCAATC | Mutation of K85 and Q86 to alanine |
| Arid1a_core_fw_Gibson | GAGAATCTTTATTTTCAGGGCGAACCGGAACGCAAAATGTGGGT | Gibson assembly Arid1a ARID in pET-Trx1a backbone |
| Arid1a_core_rev_Gibson | CGGGCTTTGTTAGCAGCCGGATCCCGACCCTTAATCTTCGCCACGTTCGATTTTG |  |
| Arid5b_core_fw | GAGAATCTTTATTTTCAGGGCCGTGCGGA  TGAACAAGCCTTTC | Gibson assembly Arid5b ARID in pET-Trx1a backbone |
| Arid5b_core_rev | CGGGCTTTGTTAGCAGCCGGATCCCGAC  CCTTAGTCCTCTTCGCCTTTGATGAAGC |  |
| JARID1a_core_fw | GAGAATCTTTATTTTCAGGGCACACGTGT  TCGCCTTGATTTCC | Gibson assembly JARID1a ARID in pET-Trx1a backbone |
| JARID1a_core_rev | CGGGCTTTGTTAGCAGCCGGATCCCGAC  CCTTACAGCGAAACTCCACTCTGAAAC |  |
| iCLIPgibA | CTAGCATGGCAGCACCGCCCGCGAAAG | Gibson assembly mArid5a in pEGFP-N1 |
| iCLIPrevBamHI | (GGATCCTCGCTAGGCTCTAGAGCTGCCACCTCGAGATACAGTTTGGTGTTC | Gibson assembly mArid5a in pEGFP-N1 |
| iCLIPgibD | GCAGCTCTAGAGCCTAG | Amplification of pEGFP-N1 for Gibson assembly |
| iCLIPgibC | GCCATGCTAGCGGATCTGACGGTTCAC | Amplification of pEGFP-N1 for Gibson assembly |
| Arid-for-StrepN.fwd | GGCTACggtctcCCGGACGCGAGGAAGAGCAGGAAC | Amplification of Arid5A_49-152 for Golden Gate Cloning |
| Arid-for-StrepN.rev | GGCTACggtctcCGTTATTTGTCATCTTCGCCTTTCAG | Amplification of Arid5A_49-152 for Golden Gate Cloning |

## Supplementary Table 2. Vectors used in this study.

| Name | Feature/Background etc. | Application |
| --- | --- | --- |
| pET-Trx | N-terminal His_6_-tag, Thioredoxin-tag, TEV cleavage site, Kanamycin resistance, *lac* repressor, T7 promotor | Cloning and expression of ARID constructs in *E. coli* |
| pEGFP-N1 | N-terminal GFP tag, Neomycin/Kanamycin resistance, CMV promotor | Expression of GFP-tagged murine fl-Arid5a in P19 cells |
| sPHDV64 | Ampicillin resistance, T7 promotor, 3’-HDV ribozyme | *In vitro* transcription of RNAs |
| pET_TRX_Bsa_  StrepTag-N | N-terminal His_6_-tag, Thioredoxin-tag, TEV cleavage site, Twin-Strep tag, Kanamycin resistance, *lac* repressor, T7 promotor | Cloning and expression of RBNS-ARID construct in *E. coli* |

## Supplementary Table 3: Overview of DNA oligonucleotides used for binding studies with ARIDs.

| Name | 5’ modification | 5’-3’ Sequence | Usage |
| --- | --- | --- | --- |
| FAM-13merAT_fw | Fluorescein | [FAM] GCACAATATAACG | F-EMSA |
| 13merAT_fw |  | GCACAATATAACG | NMR |
| 13merAT_rev |  | CGTTATATTGTGC | NMR/F-EMSA |
| FAM-13merGC_fw | Fluorescein | [FAM] GCCCCGCCCGCCG | F-EMSA |
| 13merGC_fw |  | GCCCCGCCCGCCG | NMR |
| 13merGC_rev |  | CGGCGGGCGGGGC | NMR/F-EMSA |
| FAM-13merAT_var1_fw | Fluorescein | [FAM] GCGCAATATAGCG | F-EMSA |
| 13merAT_var1_rev |  | CGCTATATTGCGC | F-EMSA |
| FAM-13merAT_var2_fw | Fluorescein | [FAM] GCACGGTATAACG | F-EMSA |
| 13merAT_var2_rev |  | CGTTATACCGTGC | F-EMSA |
| FAM-13merAT_var3_fw | Fluorescein | [FAM] GCACAACGTAACG | F-EMSA |
| 13merAT_var3_rev |  | CGTTACGTTGTGC | F-EMSA |
| FAM-13merAT_var4_fw | Fluorescein | [FAM] GCACAATACGACG | F-EMSA |
| 13merAT_var4_rev |  | CGTCGTATTGTGC | F-EMSA |
| FAM-13merAT_var5_fw | Fluorescein | [FAM] GCACAACACAACG | F-EMSA |
| 13merAT_var5_rev |  | CGTTGTGTTGTGC | F-EMSA |

## Supplementary Table 4. Overview of oligonucleotides for generating IVT templates

| Name | Sequence 5’ → 3’ | Usage |
| --- | --- | --- |
| T7fw_IVT | ACGAATTCTAATACGACTCACTATA | PCR amplification of product for IVT |
| T7rev-HDV-IVT | CGGGAGGATCCGAGCTTGTCCC | PCR amplification of product for IVT |
| 19mer_top_fw | ATACGACTCACTATAGGCACAATATAACGTCGCCGCCGGCCATGGTCCC | GA of 19mer_top into sPHDV64 |
| 19mer_top_rev | GGGACCATGGCCGGCGGCGACGTTATATTGTGCCTATAGTGAGTCGTAT | GA of 19mer_top into sPHDV64 |
| 19mer_bottom_fw | ATACGACTCACTATAGGCGACGTTATATTGTGCCGCCGGCCATGGTCCC | GA of 19mer_bottom into sPHDV64 |
| 19mer_bottom_rev | GGGACCATGGCCGGCGGCACAATATAACGTCGCCTATAGTGAGTCGTAT | GA of 19mer_bottom into sPHDV64 |
| Il6_fw^(a)^ | ATACGACTCACTATAGGCACAGAACTTATGTTGTTCTCTATGGAGAACTAAAAGTATGAGCGTTAGGACACTATTTTAATTATTT | GA of Il6 3’UTR hub into sPHDV64 |
| Il6_rev^(a)^ | GGGACCATGGCCGGTATAAATATGACTTACATAAATTAACTCAGCTTCACATATTTAAATATTAATAAATTAAAAATAATTAAAATAGTG | GA of Il6 3’UTR hub into sPHDV64 |

^[a]^ Gap-filling upon annealing the reverse and forward oligonucleotides with Klenow fragment (DNA Ploymerase I, Promega) following the protocol according to manufacturer.

**Supplementary Table 5. Overview of additional NA oligonucleotides as templates for ARID proteins used in this study.** All oligonucleotides were used as non-labelled variants or ^32^P-labelled in house.

| Name | Sequence 5’ → 3’ | Usage |
| --- | --- | --- |
| **DNAs** |  |  |
| dsDNA_95mer_fw | ACGAATTCTAATACGACTCACTATAGGGACACTATTTTAATTATTTTTAATTTATTAATATTTAAATATGTGAAGCGCCGGCCATGGTCCCAGCC | Titration to ARID_37-183_ |
| dsDNA_95mer_rev | GGCTGGGACCATGGCCGGCGCTTCACATATTTAAATATTAATAAATTAAAAATAATTAAAATAGTGTCCCTATAGTGAGTCGTATTAGAATTCGT | Titration to ARID_37-183_ |
| dsDNA_70mer_fw | GAATTCTAATACGACTCACTATAGCATTACTACAGGTTCGCCTGCCTGGTGGTGCGCCGGCCATGGTCCC | Titration to ARID_37-183_ |
| dsDNA_70mer_rev | GGGACCATGGCCGGCGCACCACCAGGCAGGCGAACCTGTAGTAATGCTATAGTGAGTCGTATTAGAATTC | Titration to ARID_37-183_ |
| dsDNA_21mer_fw | GAATGCACTGGGCCTGTTTCC | Titration to ARID_37-183_ |
| dsDNA_21mer_rev | GGAAACAGGCCCAGTGCATTC | Titration to ARID_37-183_ |
| **RNAs** |  |  |
| 15nt | GGCCUCUGUGAGGCC | rEMSA with ARID_37-183_ |
| 64nt | GUGUGGCUGUCACUCGGCUGCAUGCUUAGUGCACUCACGCAGUAUAAUUAAUAACUAAUUACUG | rEMSA with ARID_37-183_ and _49-152_ |
| 150nt | GGUCGUUGACAGGACACGAGUAACUCGUCUAUCUUCUGCAGGCUGCUUACGGUUUCGUCCGUGUUGCAGCCGAUCAUCAGCACAUCUAGGUUUCGUCCGGGUGUGACCGAAAGGUAAGAUGGAGAGCCUUGUCCCUGGUUUCAACGAGAA | rEMSA with ARID_37-183_ |
| 129_Il6_3’UTR hub | GGCACAGAACUUAUGUUGUUCUCUAUGGAGAACUAAAAGUAUGAGCGUUAGGACACUAUUUUAAUUAUUUUUAAUUUAUUAAUAUUUAAAUAUGUGAAGCUGAGUUAAUUUAUGUAAGUCAUAUUUAUA | Titration to ARID_37-183_ |
| 60mer_Il6 | GGACACUAUUUUAAUUAUUUUUAAUUUAUUAAUAUUUAAAUAUGUGAAGCUGAGUUAAUU | Titration to ARID_37-183_ |
| 19mer_ADE | CACACCGUUCUAGGUGCUG | Titration to ARID_37-183_ |
| 19mer_top | GGCACAAUAUAACGUCGCC | Titration to ARID constructs |
| 19mer_bottom | GGCGACGUUAUAUUGUGCC | Titration to ARID constructs |
| RBNS-9mer-fw | ACAGGCAGG | Titration to ARID_49-152_ |
| RBNS-9mer-rev | CCUGCCUGU | Titration to ARID_49-152_ |

**Supplementary Table 6. Amino acid sequences of the proteins used for RBNS assay.** The Twin-Strep-tag is marked in bold.

| **Name** | **Amino acids** |
| --- | --- |
| ARID_49-152_ with TRX | HHHHHHPMSDKIIHLTDDSFDTDVLKADGAILVDFWAEWCGPCKMIAPILDEIADEYQGKLTVAKLNIDQNPGTAPKYGIRGIPTLLLFKNGEVAATKVGALSKGQLKEFLDANLAGSGSGSENLYFQGAMAREEEQEREEEQAFLVSLYKFMKERHTPIERVPHLGFKQINLWKIYKAVEKLGAYELVTGRRLWKNVYDELGGSPGSTSAATCTRRHYERLVLPYVRHLKGEDDK |
| Twin-Strep-tagged ARID_49-152_ with TRX | HHHHHHPMSDKIIHLTDDSFDTDVLKADGAILVDFWAEWCGPCKMIAPILDEIADEYQGKLTVAKLNIDQNPGTAPKYGIRGIPTLLLFKNGEVAATKVGALSKGQLKEFLDANLAGSGSGSENLYFQG**SAWSHPQFEKGGGSGGGSGGSAWSHPQFEK**SGREEEQEREEEQAFLVSLYKFMKERHTPIERVPHLGFKQINLWKIYKAVEKLGAYELVTGRRLWKNVYDELGGSPGSTSAATCTRRHYERLVLPYVRHLKGEDDK |
| TEV cleaved Twin-Strep-tagged ARID_49-152_ | G**SAWSHPQFEKGGGSGGGSGGSAWSHPQFEK**SGREEEQEREEEQAFLVSLYKFMKERHTPIERVPHLGFKQINLWKIYKAVEKLGAYELVTGRRLWKNVYDELGGSPGSTSAATCTRRHYERLVLPYVRHLKGEDDK |

**Supplementary Table 7. Oligonucleotides used for the RBNS assay.**

| **Name** | **Sequences 5' → 3'** |
| --- | --- |
| T7 promoter oligo | GTATAATACGACTCACTATAGGG |
| RBNS T7 template (20mer) | CCTTGGCACCCGAGAATTCCA(N)_20_GATCGTCGGACTGTAGAACTCCCTATAGTGAGTCGTATTATAC |
| RNA RT Primer (RTP) | GCCTTGGCACCCGAGAATTCCA |
| RNA PCR Primer (RP1) | AATGATACGGCGACCACCGAGATCTACACGTTCAGAGTTCTACAGTCCGACGATC |
| RNA PCR Index 1 (RPI1) | CAAGCAGAAGACGGCATACGAGAT**CGTGAT**GTGACTGGAGTTCCTTGGCACCCGAGAATTCCA |
| RNA PCR Index 2 (RPI2) | CAAGCAGAAGACGGCATACGAGAT**ACATCG**GTGACTGGAGTTCCTTGGCACCCGAGAATTCCA |
| RNA PCR Index 3 (RPI3) | CAAGCAGAAGACGGCATACGAGAT**GCCTAA**GTGACTGGAGTTCCTTGGCACCCGAGAATTCCA |
| RNA PCR Index 4 (RPI4) | CAAGCAGAAGACGGCATACGAGAT**TGGTCA**GTGACTGGAGTTCCTTGGCACCCGAGAATTCCA |

**Supplementary Table 8. Indices used for Illumina sequencing.**

| **Sample Name** | **i7 Index Name** | **i7 Index Sequence** |
| --- | --- | --- |
| input | RPI1 | ATCACG |
| ARID-0.25-µM | RPI2 | CGATGT |
| ARID-1-µM | RPI3 | TTAGGC |
| ARID-5-µM | RPI4 | TGACCA |

**Supplementary Table 9. Primers and indices used for iCLIP2.**

| **Name** | **Sequences 5' → 3'** |
| --- | --- |
| Lclip2.0 adapter Rep 1 | P-NNNN**ACATCG**NNNNNAGATCGGAAGAGCGTCGTG-ddC |
| Lclip2.0 adapter Rep 2 | P-NNNN**GCCTAA**NNNNNAGATCGGAAGAGCGTCGTG-ddC |
| Lclip2.0 adapter Rep 3 | P-NNNN**CACTGT**NNNNNAGATCGGAAGAGCGTCGTG-ddC |
| L3- App | rApp-AGATCGGAAGAGCGGTTCAG-ddC |
| Solexa short P3 | CTGAACCGCTCTTCCGATCT |
| Solexa short P5 | ACACGACGCTCTTCCGATCT |
| Solexa long P3 | CAAGCAGAAGACGGCATACGAGATCGGTCTCGGCATTCCTGCTGAACCGCTCTTCCGATCT |
| Solexa long P5 | AATGATACGGCGACCACCGAGATCTACACTCTTTCCCTACACGACGCTCTTCCGATCT |
| RT primer | GGATCCTGAACCGCT |


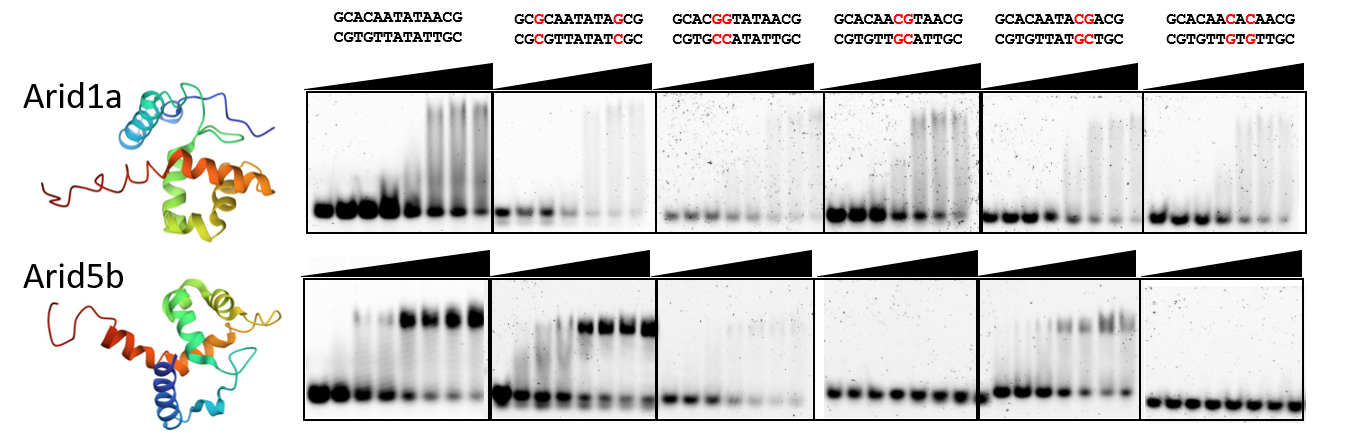


**Supplementary Fig. 1. DNA-preferences of selected ARID domains.** EMSAs of fluorescently labelled DNAs (10 nM) as given above when titrated with increasing amounts of extended ARID domains from either Arid1a (top) or Arid5b (bottom). Protein concentrations were 0, 1, 2, 3, 5, 10, 15, 30 and 0, 0.5, 0.75, 1, 1.5, 2, 3, 5 µM for Arid1a and Arid5b, respectively. All experiments have been carried out in standard Arid5a buffer.


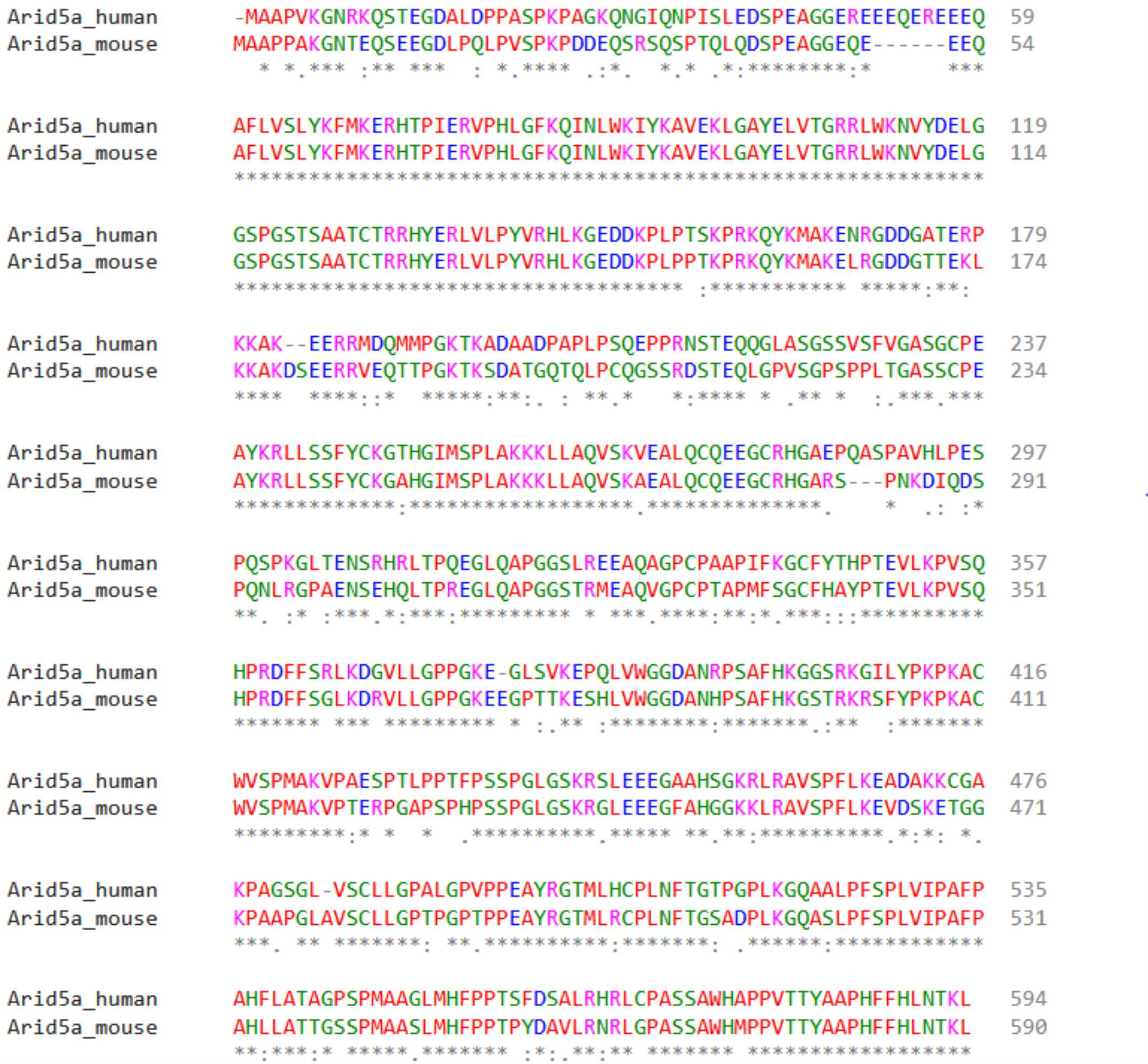


**Supplementary Fig. 2.** **Comparison of complete human and mouse Arid5a protein sequences** based on UniProt accession codes Q03989 and Q3U108, respectively. Alignment created with Clustal Omega^1^.


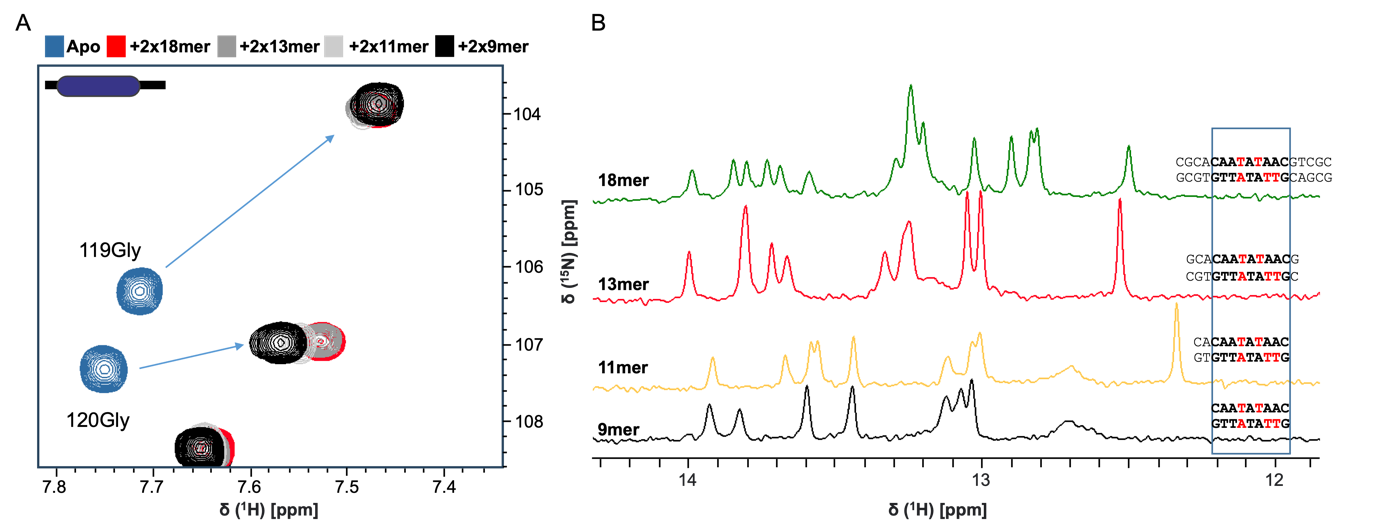
a) b)

**Supplementary Fig. 3. Finding the minimal dsDNA length for ARID domain binding. a)** ^1^H-^15^N HSQC spectra of various dsDNA lengths in 2-fold excess to ARID_37-183_ (color code indicated above). **b)** Imino-proton spectra of dsDNAs used in a) show the integrity of duplexed DNA species. Sequences are given, the central AT-rich motif is boxed. All experiments have been carried out in standard Arid5a buffer.


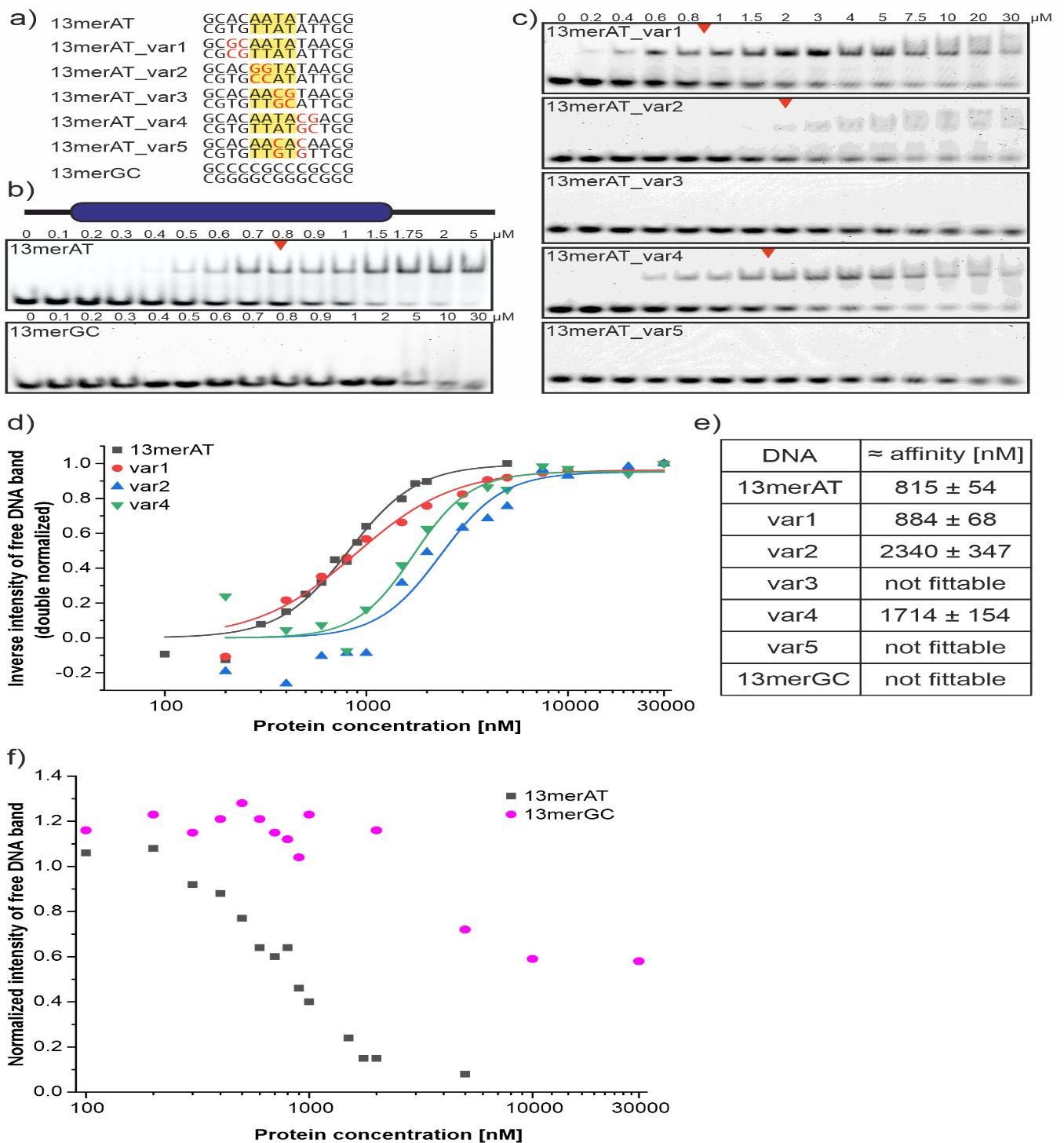


**Supplementary Fig. 4**: **Arid5a ARID domain prefers AT-rich DNA.** **a)** Sequences of DNA variants used for EMSAs. The central AT-rich motif is highlighted in yellow. Nucleotide exchanges in variants are colored red. Note that 13mer_AT_var2 still provides a stretch of 5A/Ts despite the placement of GC in its central, highlighted region. **b)** EMSAs, carried out in standard Arid5a buffer, showing ARID_37-183_ discriminating between AT-rich and GC-rich dsDNA. EMSAs were run with 5'-fluorescently labelled dsDNA (10 nM) and increasing amounts of protein, as indicated above. **c)** EMSAs showing ARID_37-183_ discriminating between the 13merAT-variants. In b) and c), red arrows indicate K_D_ values obtained from d). **d)** Quantification of free DNA from b) and c). The quantification of band intensity was done in ImageQuant TL and values normalized to the free DNA band at 0 µM protein (0) and at the highest protein concentration (1). Normalized values are plotted as a function of protein concentration. **e)** Summary of K_D_ values based on EMSA quantification from b)-d). The errors are derived from the fits. See source data file for complete fit data. **f)** Example of raw data analysis showing the quantification of decreasing free DNA intensity from panel b) for 13merAT vs. 13merGC.


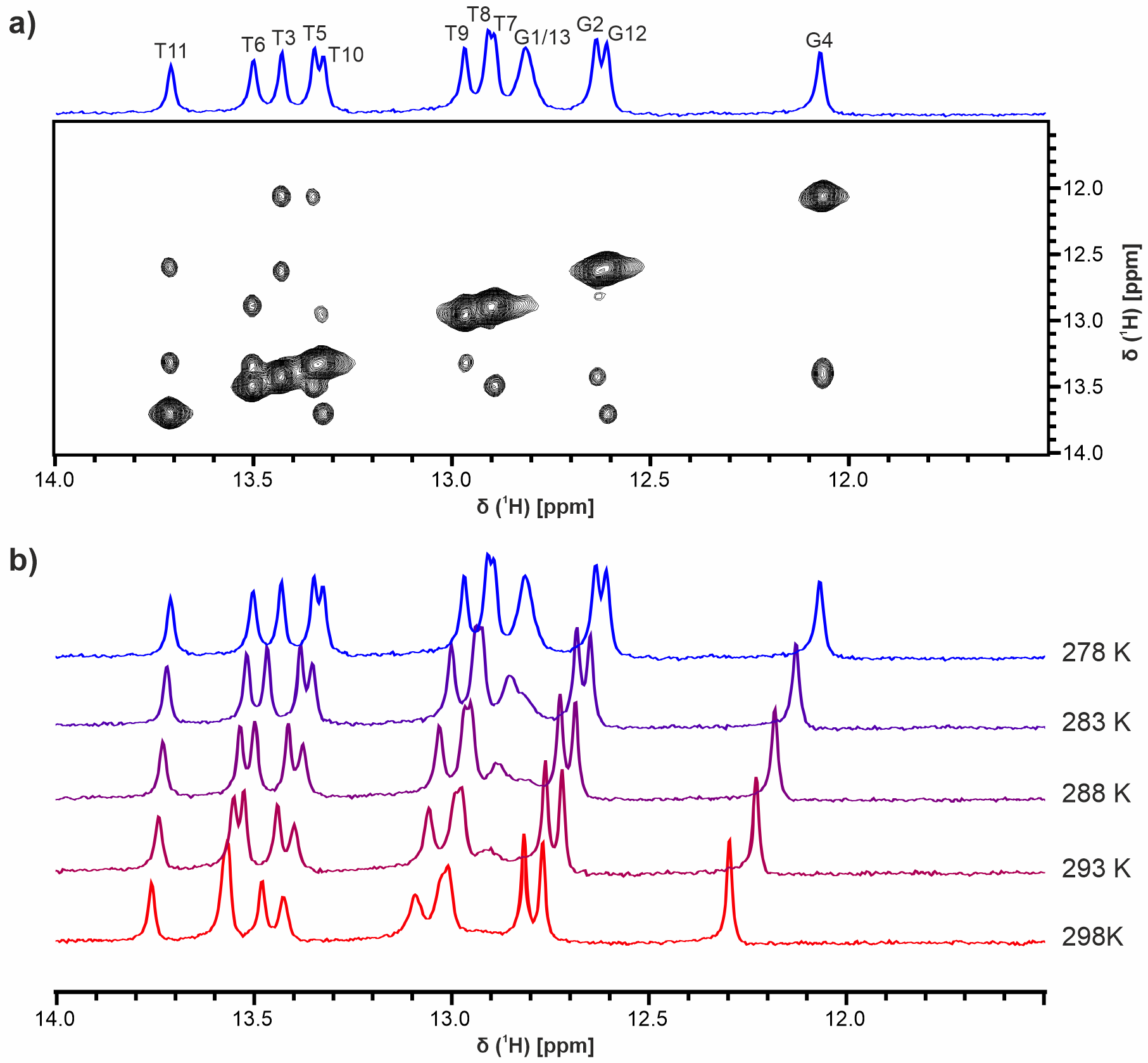


**Supplementary Fig. 5. Assignment of 13merAT DNA. a)** ^1^H-^1^H NOESY of 13merAT used to assign imino proton peaks at 278 K. **b)** Temperature series of 13merAT imino proton spectra to transfer the assignment from 278 K to 298 K (as shown in **Fig. 3**). Spectra were measured with 280 µM dsDNA at 600 MHz in standard Arid5a buffer.


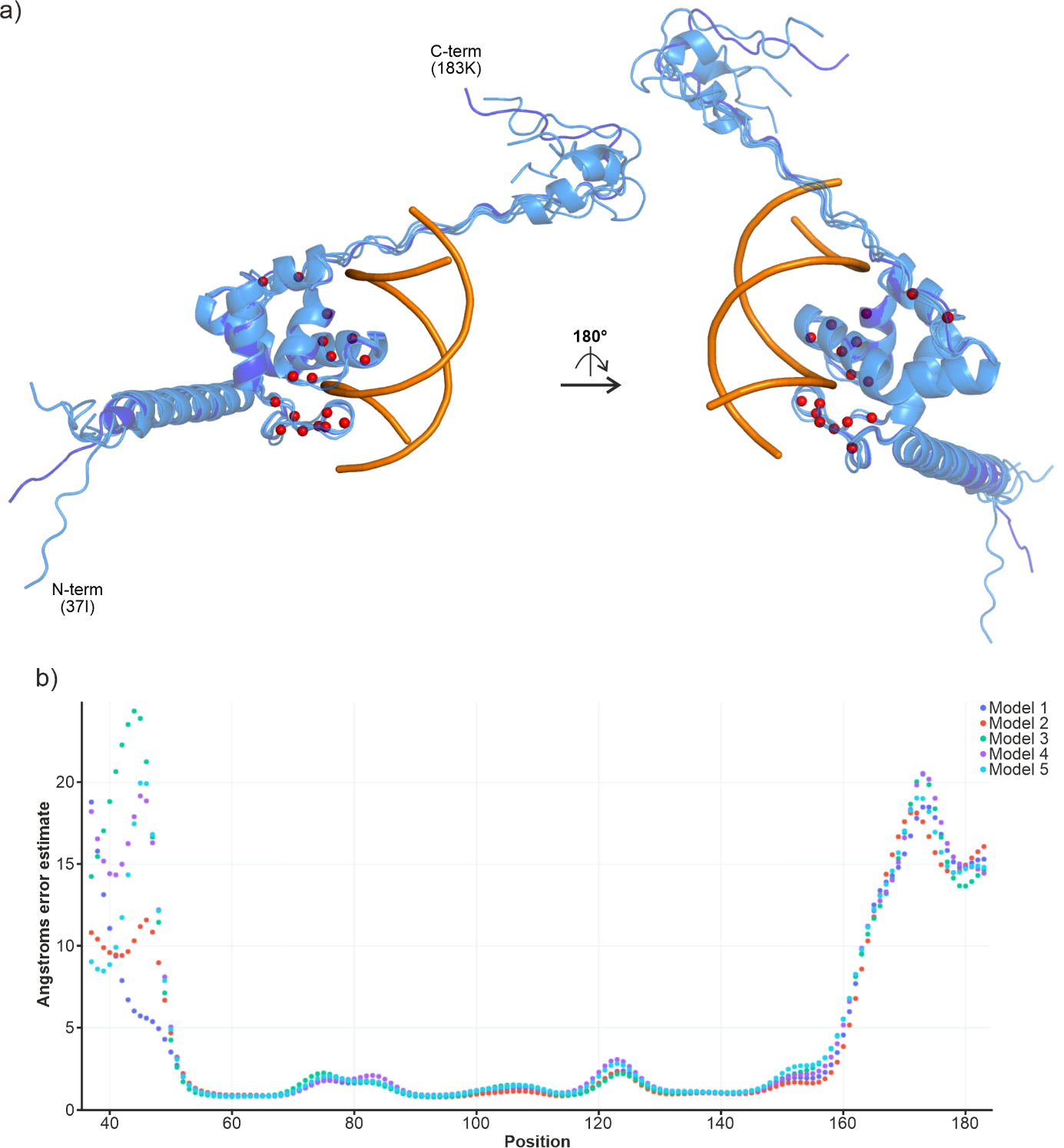


**Supplementary Fig. 6. Comparison of 5 models for ARID_37-183_.** **a)** Alignment of 5 RoseTTAFold^2^ models of ARID_37-183_. Dark blue shows the model used in **Fig. 3c,** to which the other 4 models (in light blue) have been aligned. **b)** Error estimate in angstroms as generated by RoseTTAFold^2^ for all 5 models generated. The absolute backbone r.m.s.d. values for the ensemble are 0.599 ± 0.063 Å for ARID_37-183_ and 0.499 ± 0.062 Å for ARID_49-152_.


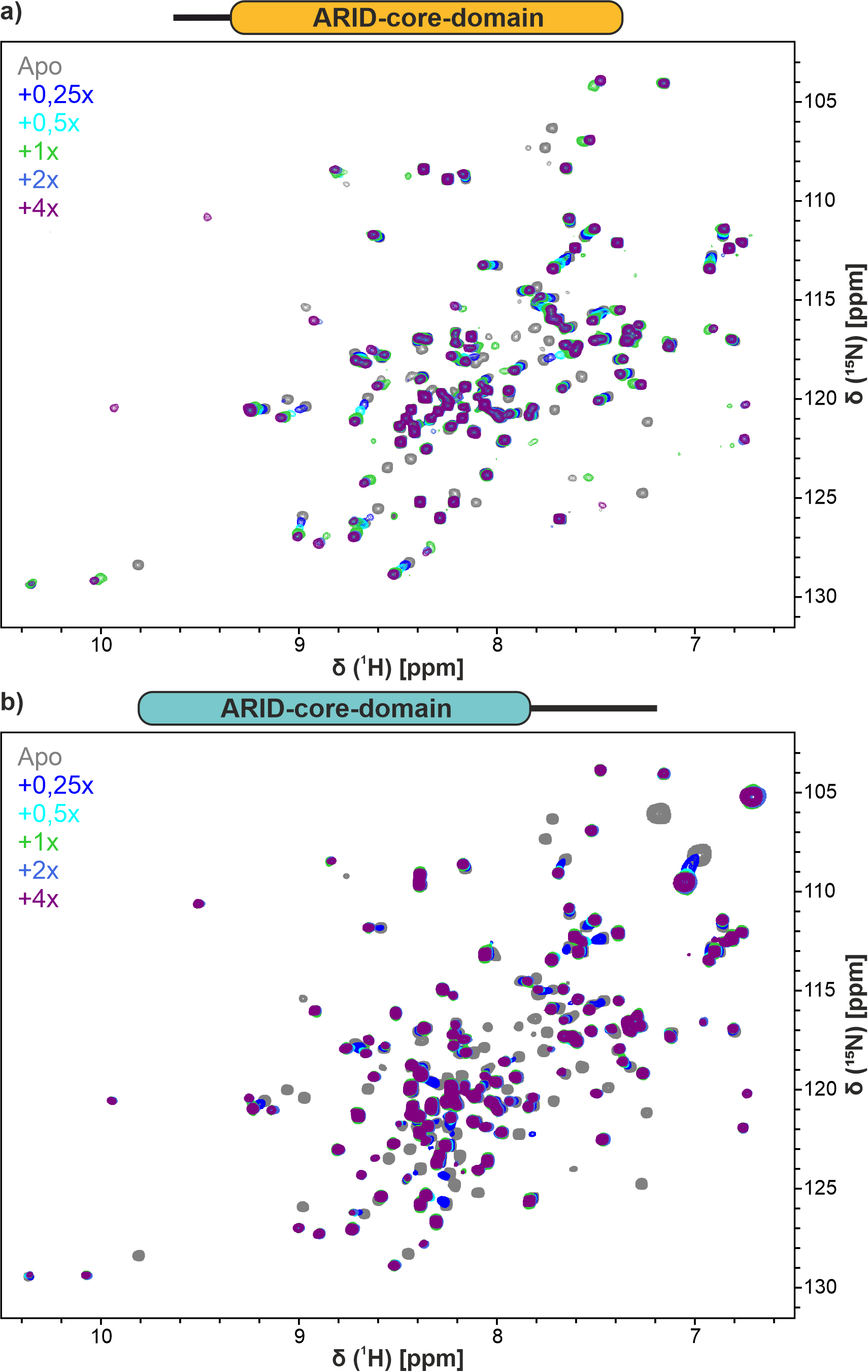


**Supplementary Fig. 7**. **NMR-observed titrations of ARID_37-152_ and _49-183_ with 13merAT.** ^1^H-^15^N HSQC spectra of ARID_37-152_ **(a)** and _49-183_ **(b)** alone (gray) and with increasing concentrations of 13merAT as indicated by the color code in upper left corner. Spectra were recorded with 70 µM protein at 600 MHz and 298 K in standard Arid5a buffer.


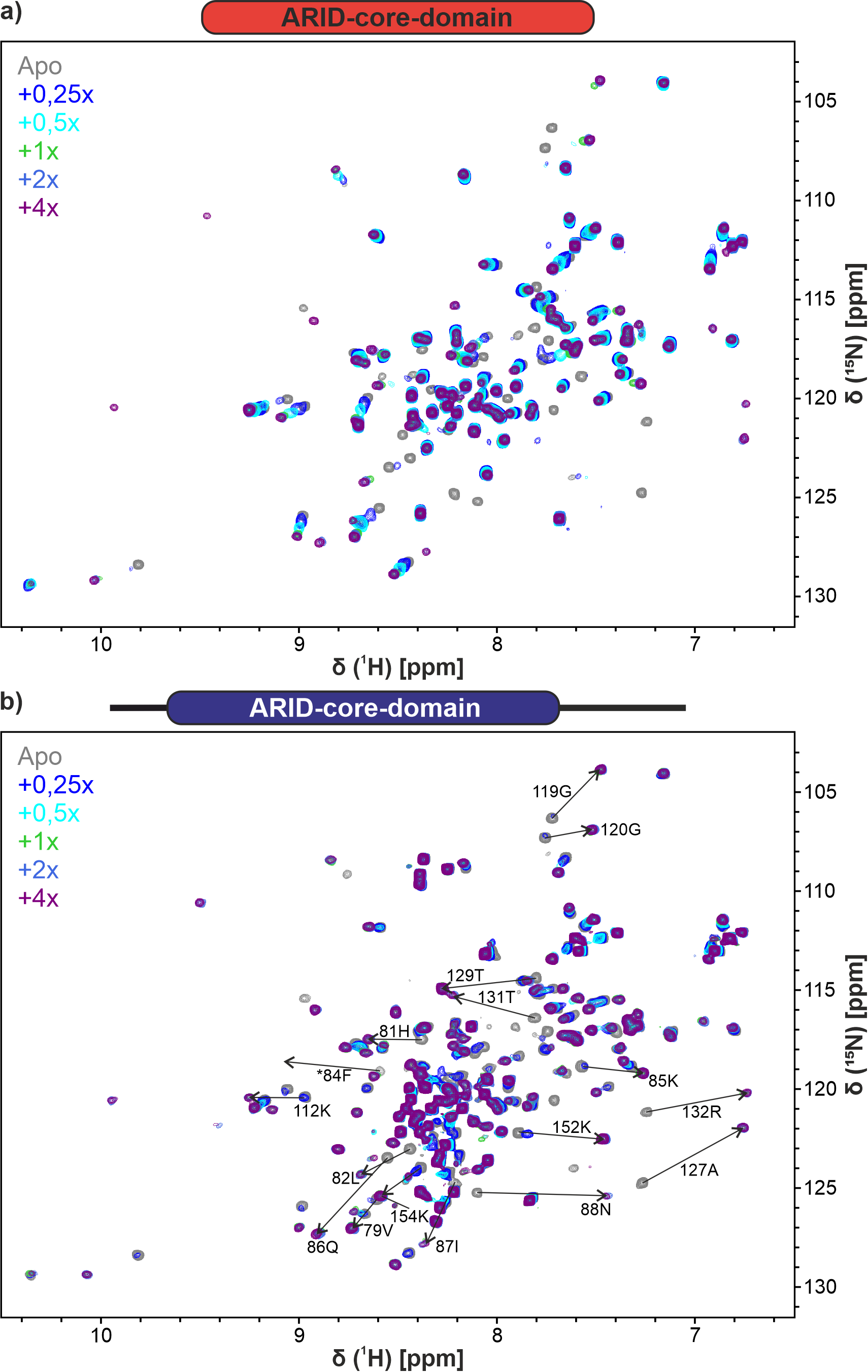


**Supplementary Fig. 8**. **NMR-observed titrations of ARID_49-152_ and _37-183_ with 13merAT.** ^1^H-^15^N HSQC spectra of ARID_49-152_ **(a)** and _37-183_ **(b)** alone (gray) and with increasing concentrations of 13merAT as indicated by the color code in upper left corner. Assignments for CSPs with values above mean+1 standard deviation (SD, according to **Fig. 3b**) are shown with respective trajectories indicated by arrows. Spectra were recorded with 70 µM protein at 600 MHz and 298 K in standard Arid5a buffer.


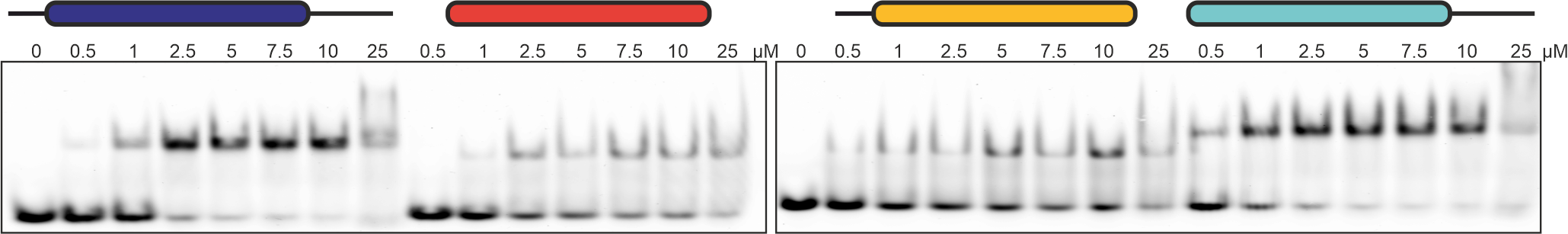


**Supplementary Fig. 9. EMSAs confirm the relative affinities of core and extended Arid5a ARID constructs** with 13merAT dsDNA (10 nM) as observed from HSQCs (Fig. 3d and Suppl. Fig. 6 and 7). EMSAs have been performed in standard Arid5a buffer.


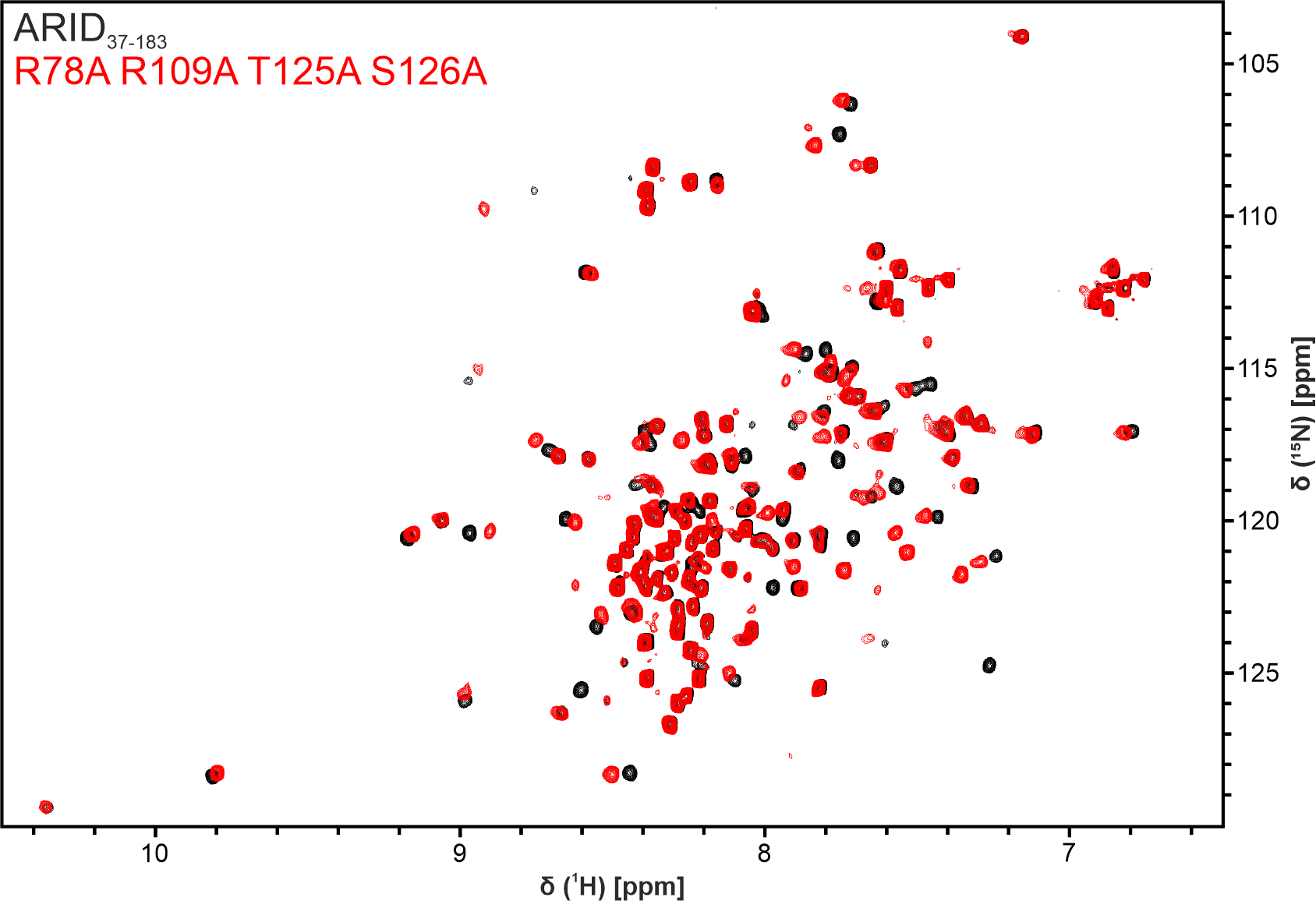


**Supplementary Fig. 10. Effect of 4x mutation within ARID domain of ARID_37-183_**. Overlay of ^1^H-^15^N HSQC spectra, recorded in standard Arid5a buffer, showing apo ARID_37-183_ WT (black) and ARID_37-183_ R78A R109A T125A S126A (red). Spectra were recorded at a protein concentration of 70 µM at 700 MHz and 298 K.


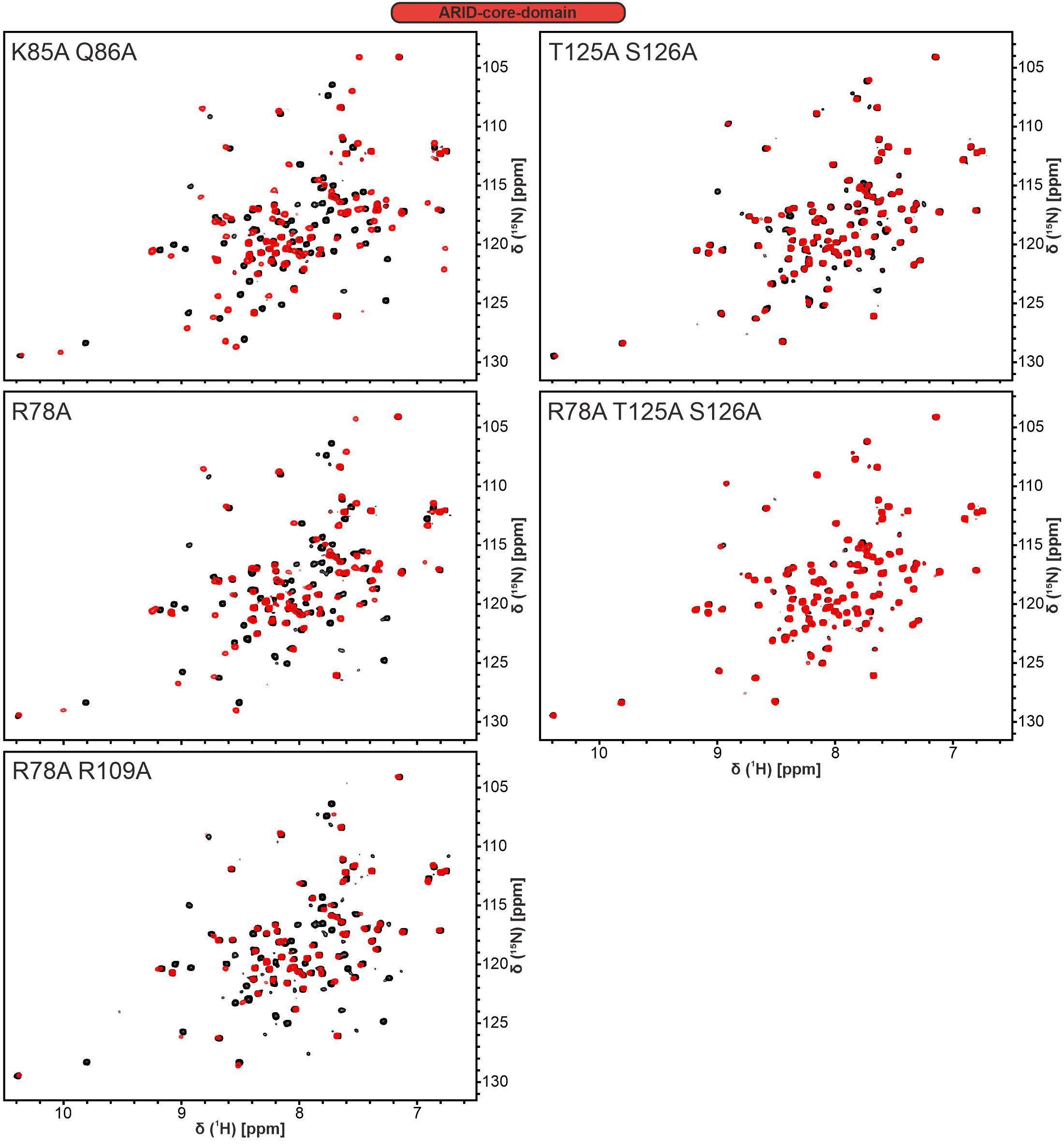


**Supplementary Fig. 11. Effects of ARID_49-152_ mutants on binding to 13merAT DNA.** Overlays of ^1^H-^15^N HSQC spectra showing ARID_49-152_ mutants as indicated in the upper left corner without (black) and with 2-fold 13merAT dsDNA (red). Spectra were recorded in standard Arid5a buffer with 70 µM protein at 600 MHz and 298 K.


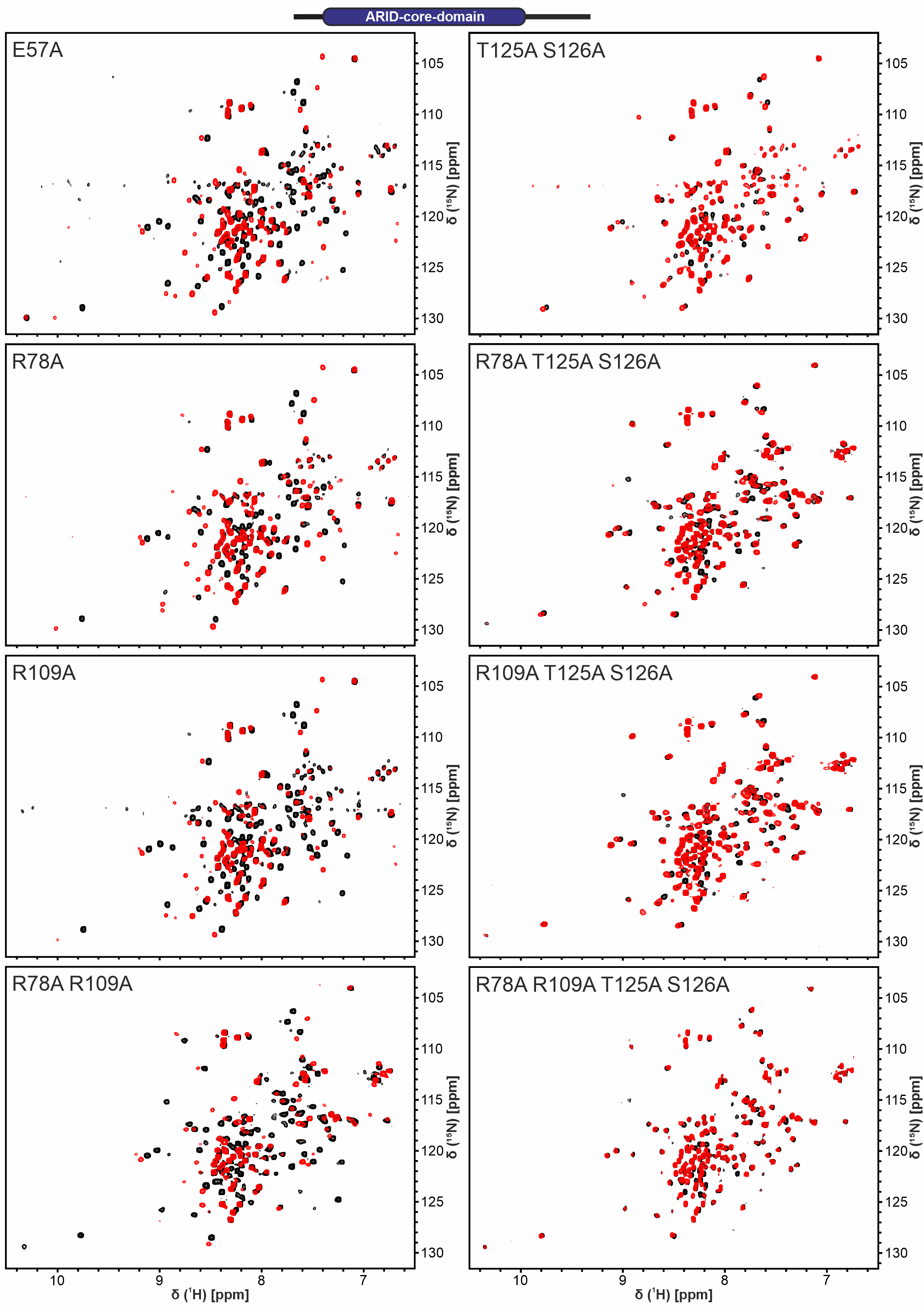


**Supplementary Fig. 12. Effects of ARID_37-183_ mutants on binding to 13merAT DNA.** Overlays of ^1^H-^15^N HSQC spectra showing ARID_37-183_ mutants as indicated in the upper left corner without (black) and with 2-fold 13merAT dsDNA (red). Spectra were recorded in standard Arid5a buffer with 70 µM protein at 600 MHz and 298 K.


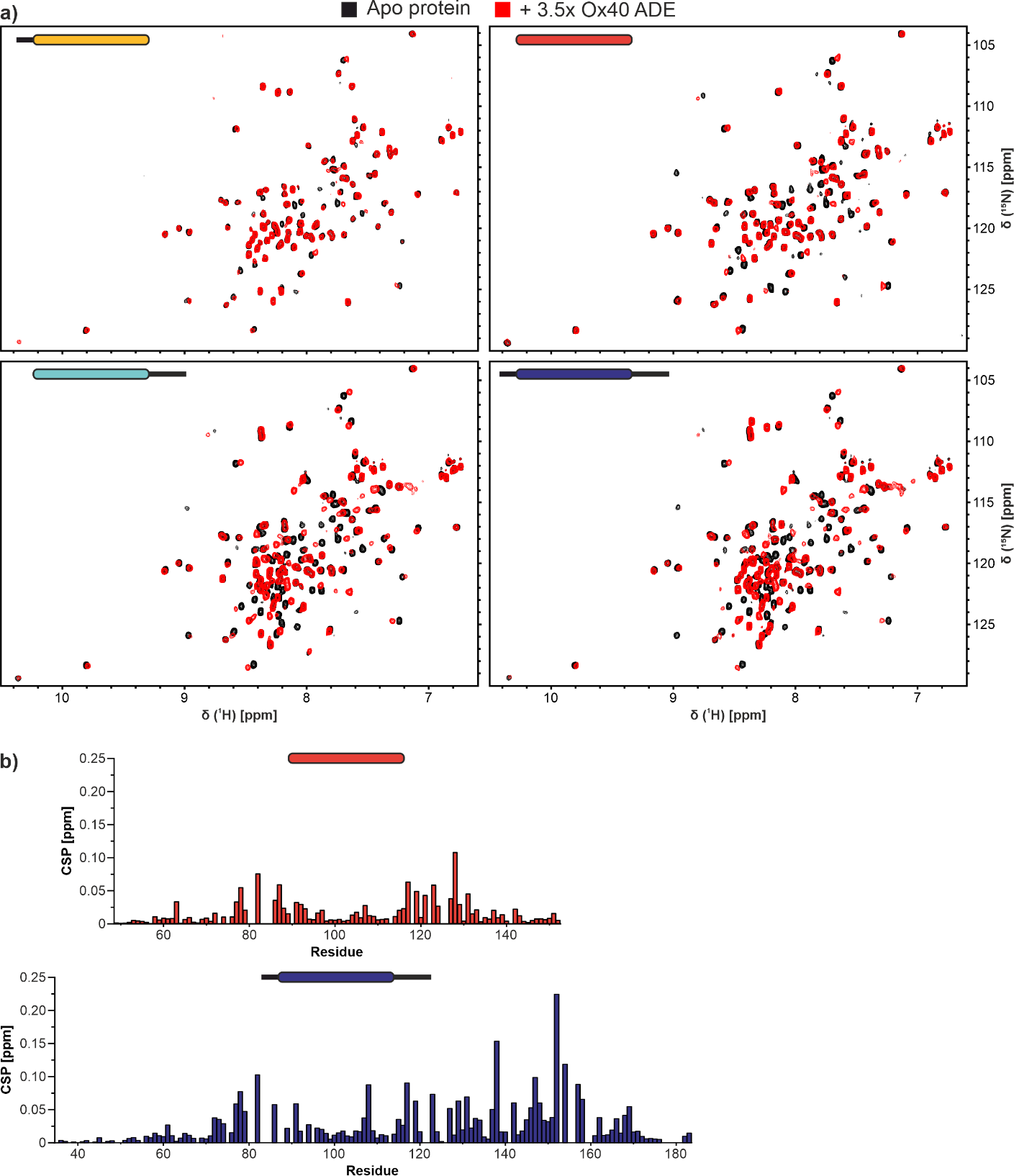


**Supplementary Fig. 13. The Arid5a ARID domain binds to the *Ox40* ADE with moderate affinity but supported by the C-terminal extension. a)** ^1^H-^15^N-HSQC spectra of apo protein (either ARID_37-152_: top left, ARID_49-152_: top right, ARID_49-183_: bottom left or ARID_37-183_: bottom right, (black)) and overlaid with the respective titration points of 3.5-fold molar excess of ADE-RNA (red). All spectra were recorded in standard Arid5a buffer with 40 µM protein at 298 K and 600 MHz. **b)** CSP plot of ARID_49-152_ (upper panel) and ARID_37-183_ (lower panel) upon titration of 3.5-fold ADE-RNA. Blank spaces in plot are either line broadened beyond detection or assignments could not be transferred unambiguously. See main text Fig. 3b for a comparison with binding to AT-DNA.


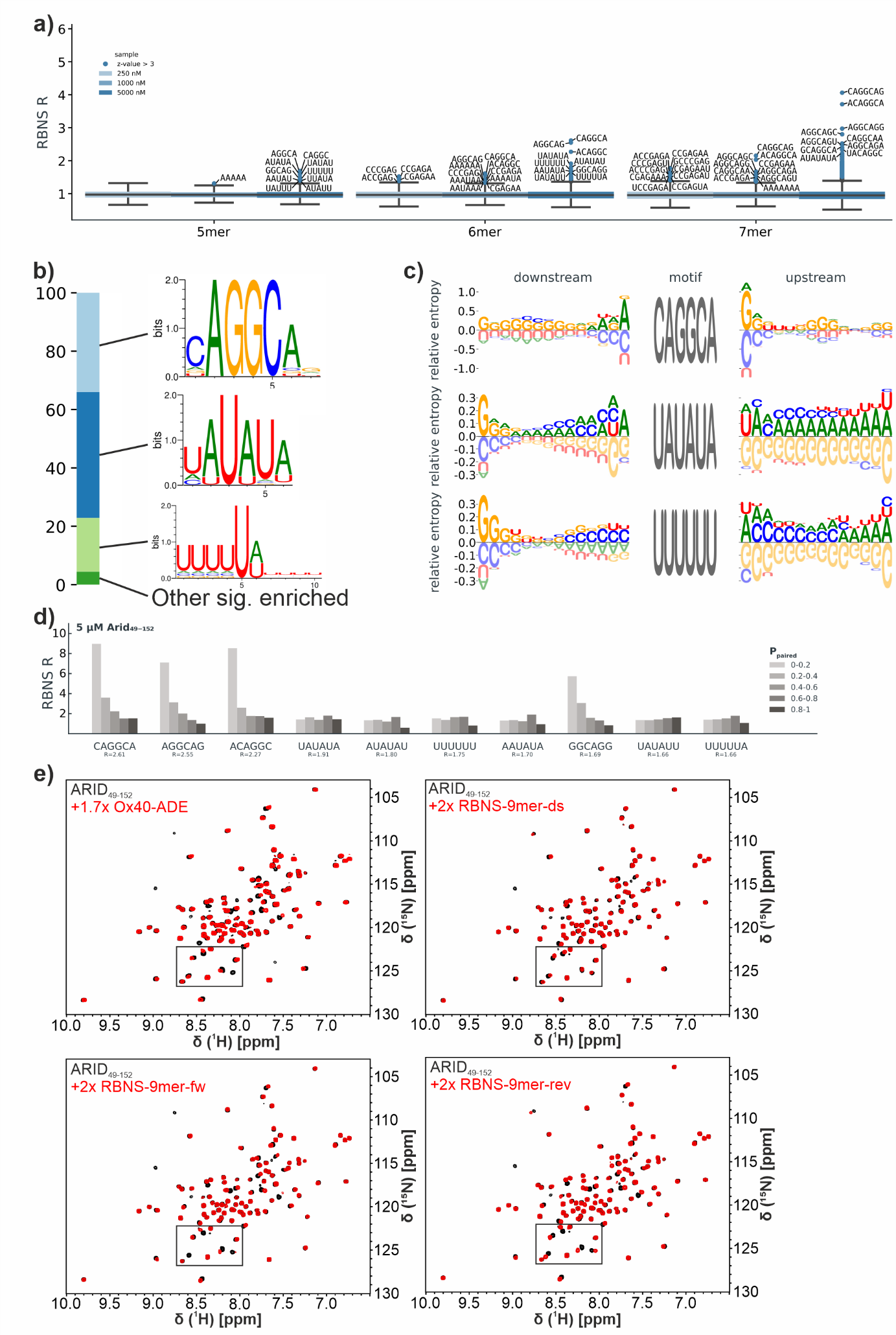


**Supplementary Fig. 14: RBNS-derived sequences for ARID_49-152_.** **a)** Enrichment of all different k-mers (k = 5,6,7) at 0.25, 1 and 5 µM RBP concentration. Values greater than three standard deviations above the mean are highlighted. For the highest significant ten motif sequences are given. Source data are provided. **b)** Significantly enriched sequences as logos in the most enriched 6-mers (z-score > 3). Bar plot is proportional to the summed k-mers. **c)** Sequence context: Search for complex binding motifs, which contain further recognition sequences in addition to selected enriched 6-mers at 5 µM ARID_49-152_ concentration. **d)** Structural features of the ten most enriched 6-mer motifs at 5 µM ARID_49-152_: The average P_paired_ value across the bases of the indicated motif was calculated for each occurrence in the library using RNAfold^3^. Motifs are sorted into five bins according to structure probability. The R value is calculated for each bin as the frequency in the pulldown library divided by that in the input library. **e)** Full view of ^1^H-^15^N-HSQC spectra of apo ARID_49-152_ overlaid with 1.7-fold molar excess of ADE RNA or 2-fold molar excess of RBNS-9mer RNAs for which zoom-ins are shown in **Fig. 5d**. NMR data were recorded in standard Arid5a buffer.


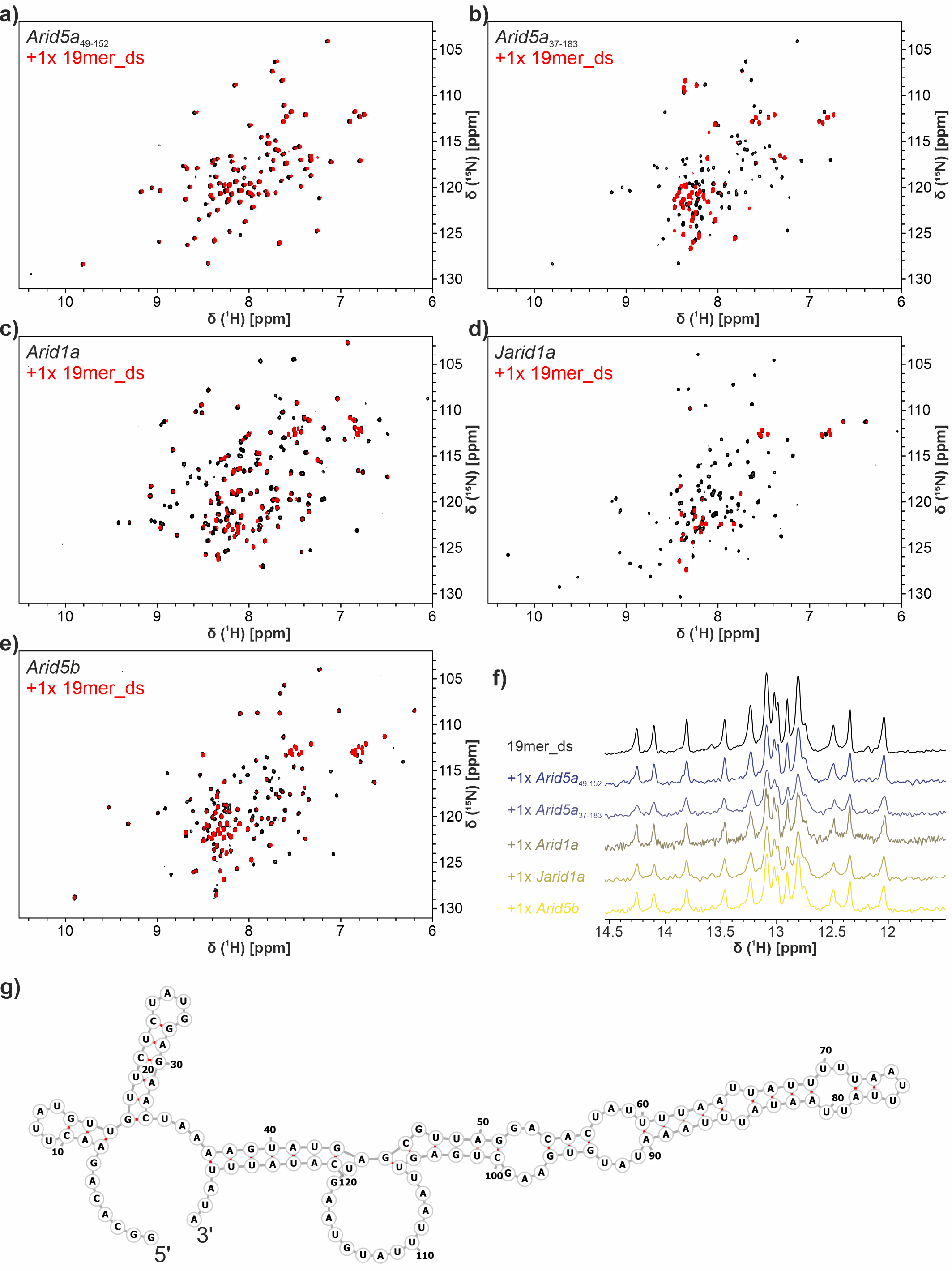


**Supplementary Fig. 15. Comparative binding of ARID domains to RNA.** **a-e)** Overlay of ^1^H-^15^N-HSQCs of Arid5a_49-152_ (a) or Arid5a_37-183_ (b), Arid1a ARID (c) Jarid1a ARID (d) and Arid5b ARID (e) without RNA (black) and with 1x 19mer_ds RNA (red). All protein concentrations were 50 µM. **f)** Comparison of imino proton spectra showing 19mer_ds DNA alone (top, black) or after addition of equimolar amounts of protein as indicated (black to yellow). RNA concentrations were 50 µM for samples with protein and 25 µM for the apo sample with the number of scans adjusted. Spectra in **a)**-**f)** were recorded in standard Arid5a buffer at 600 MHz and 298 K **g)** Vienna RNAfold^4^ RNA secondary structure predictions of the Il-6 3' UTR hub used in this study.


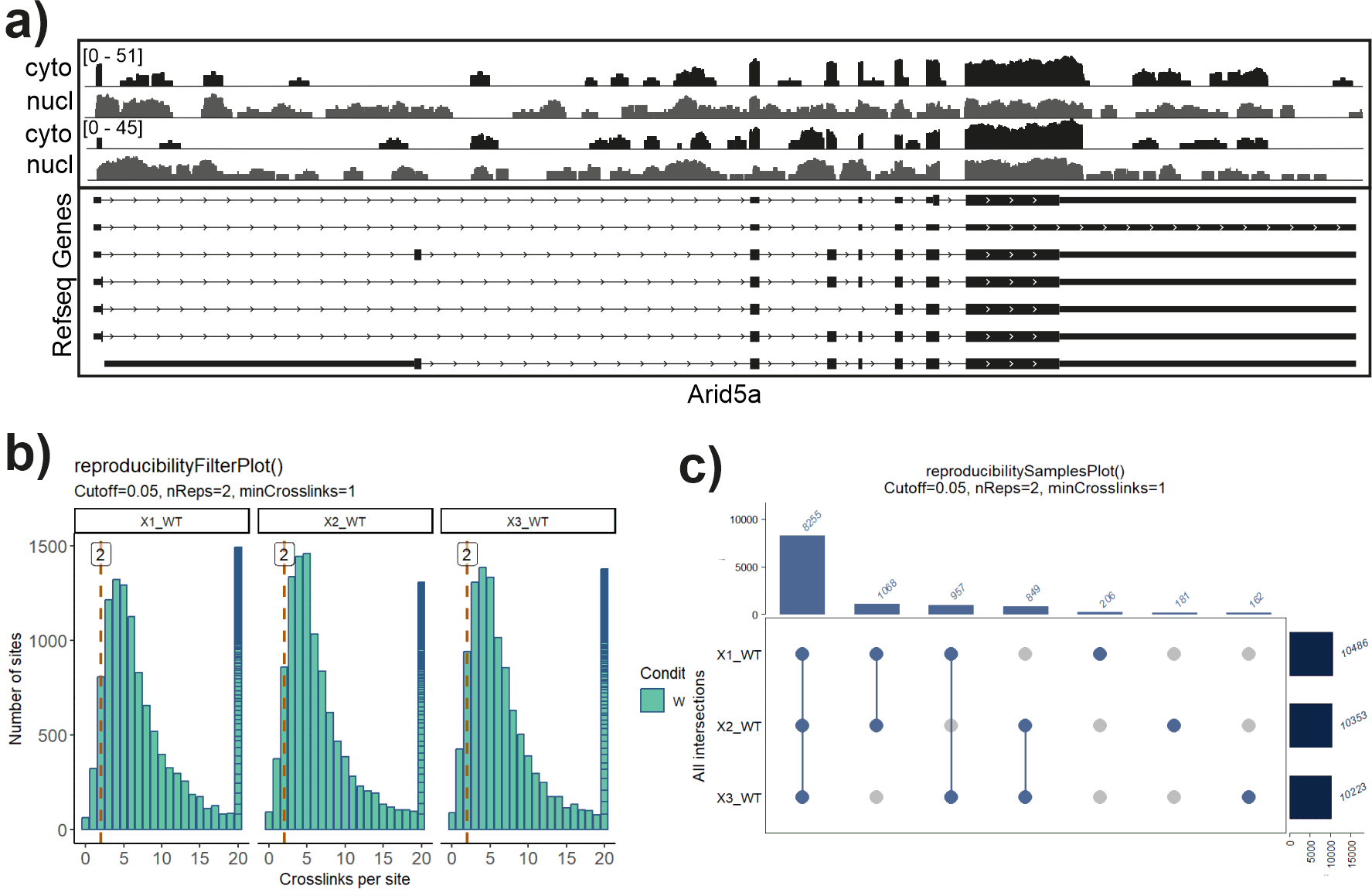


**Supplementary Fig. 16. iCLIP2 analysis of Arid5a*.* a)** Browser shot of nuclear and cytoplasmic RNAs (unpublished RNA-seq data) shows that Arid5a is expressed in murine P19 cells. **b)** Histogram shows the distribution of crosslink events per binding sites, indicating the minimum number of crosslink events that were required for a binding site to be called as present in a replicate. **c)** The vast majority of binding sites is shared by at least 2 of 3 replicates. Note that intergenic binding sites were removed later.


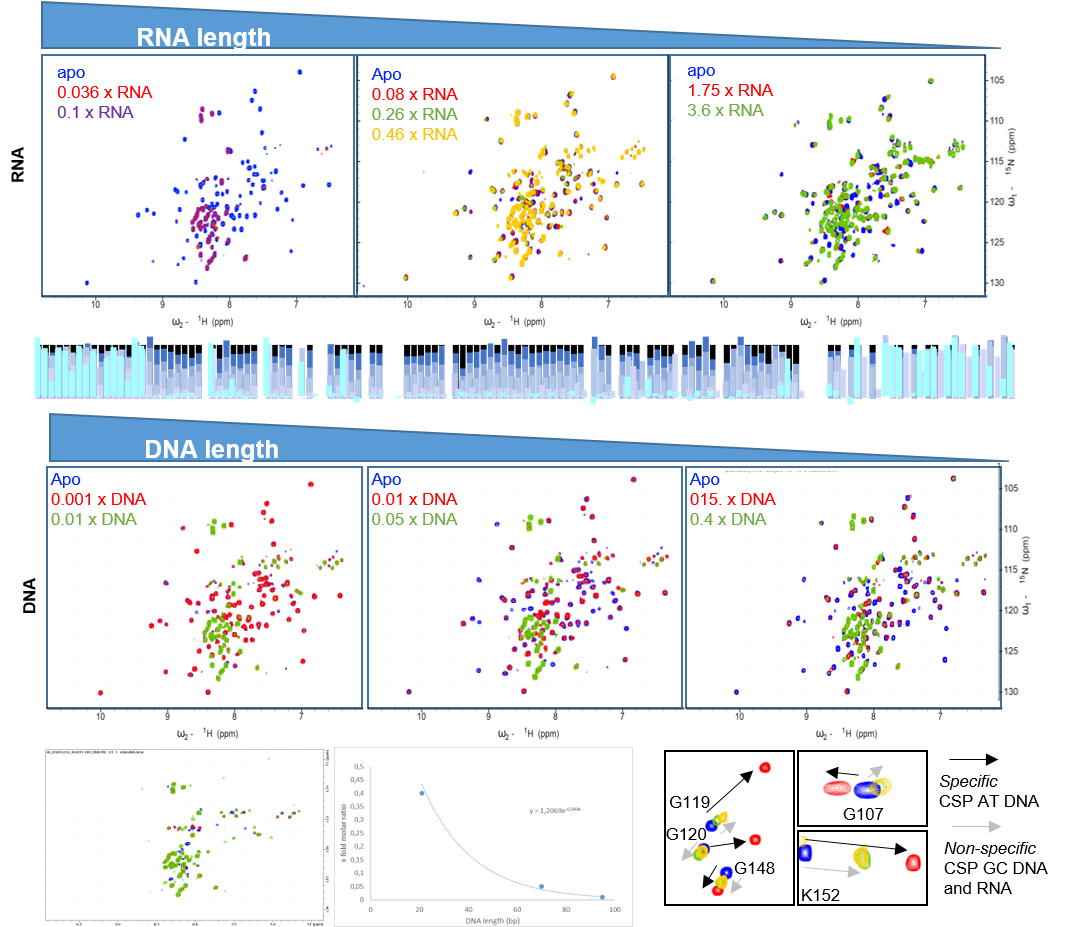


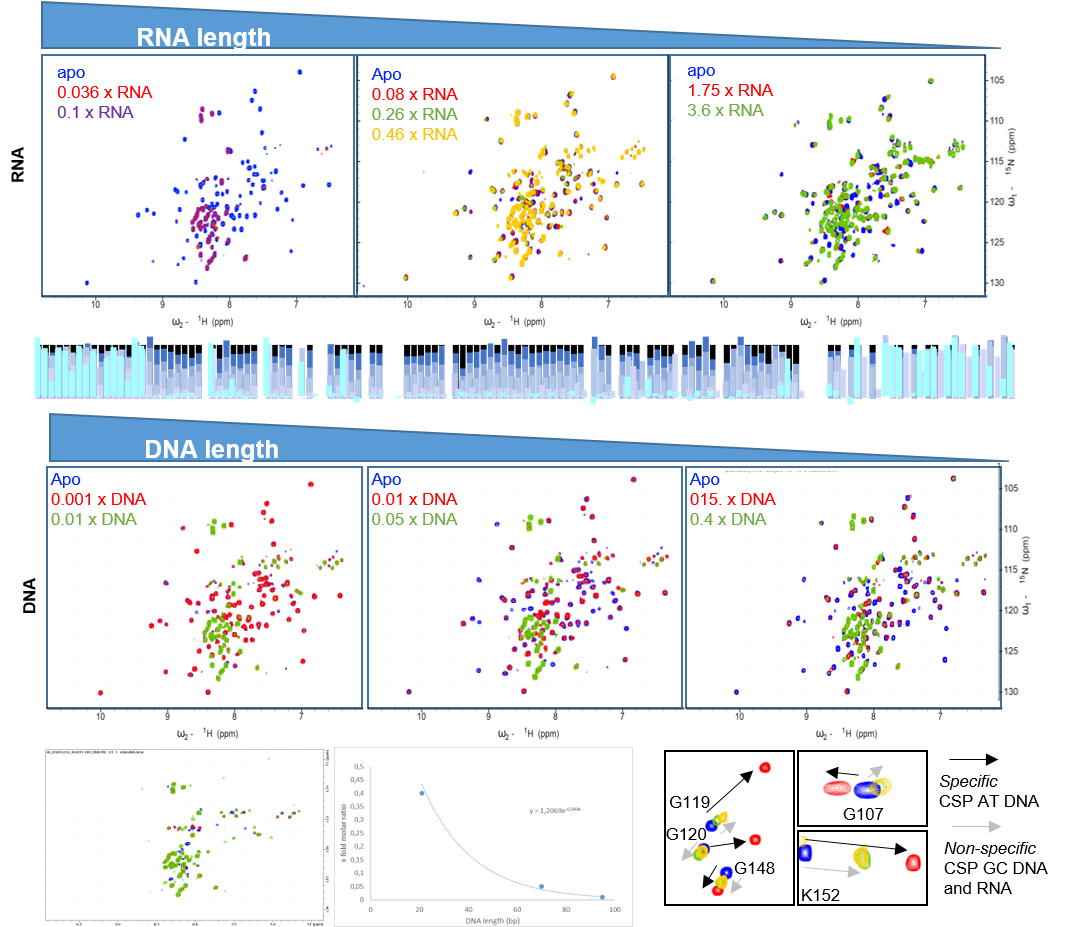


**Supplementary Fig. 17. Influence of nucleic acid-length on binding to ARID_37-183_.** Apparent affinities – as estimated by the degree of line-broadening in main text **Fig. 7** – scale with the length of nucleic acids (NAs). Molar ratios of NA equivalents are given within the spectra. Sizes of RNAs from left to right are: 129 nt (Il_6), 60 nt (Il_6_60), 19 nt (ADE); sizes of dsDNA from left to right are: 95 nt, 70 nt, 21 nt. NMR data were recorded in standard Arid5a buffer.


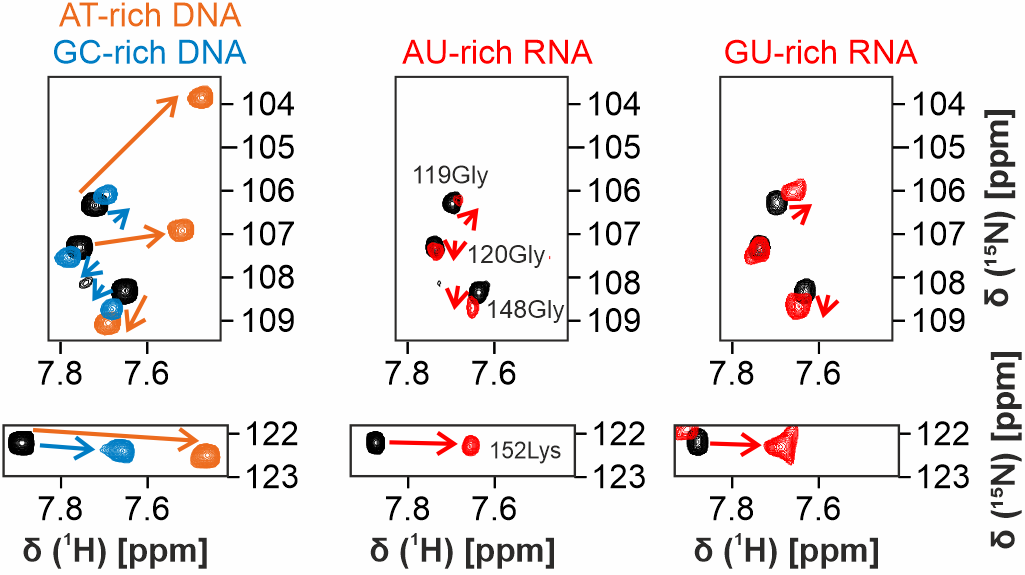


**Supplementary Fig. 18. Binding of AU/GU-rich RNA by ARID_37-183_ reveals CSPs identical with non-specific GC-rich DNA binding.** Shown is the direct comparison of respective insets from **Fig. 3a, Fig. 6b and Suppl. Fig. 11**. ^1^H-^15^N-HSQCs of ARID_37-183_ with 4x 13merAT (orange) and 13merGC (blue) DNAs as well as 1x 19mer_ds RNA (AU-rich, red) and 3.5x Ox40 ADE (GU-rich, red) are overlaid with the apo protein (black), respectively. NMR data were recorded in standard Arid5a buffer.

**Supplementary references**

1 Sievers, F. *et al.* Fast, scalable generation of high-quality protein multiple sequence alignments using Clustal Omega. *Mol Syst Biol* **7**, 539 (2011). <https://doi.org/10.1038/msb.2011.75>

2 Baek, M. Accurate prediction of protein structures and interactions using a three-track neural network. *Science* **373** (2021). <https://doi.org/10.1126/science.abj8754>

3 Lorenz, R. *et al.* ViennaRNA Package 2.0. *Algorithms Mol Biol* **6**, 26 (2011). <https://doi.org/10.1186/1748-7188-6-26>

4 Hofacker, I. L. Vienna RNA secondary structure server. *Nucleic Acids Res* **31**, 3429-3431 (2003). <https://doi.org/10.1093/nar/gkg599>
